# Strength curve data for slender geopolymer concrete columns with GFRP, steel and hybrid reinforcement

**DOI:** 10.1016/j.dib.2021.107589

**Published:** 2021-11-18

**Authors:** Mohammad AlHamaydeh, Fouad Mostafa Amin

**Affiliations:** a Professor, Department of Civil Engineering, College of Engineering, American University of Sharjah, Sharjah, PO Box 26666, UAE; b Graduate research assistant, Department of Civil Engineering, College of Engineering, American University of Sharjah, Sharjah, PO Box 26666, UAE

**Keywords:** Slender circular column, Geopolymer concrete, Glass-fiber-reinforced polymer (GFRP) bars, Hybrid reinforcement, Interaction diagram, Slenderness ratio, Strength curves

## Abstract

This article provides a wide range of circular columns strength values under different loading conditions. The provided strength values are dependent on various parameters including the longitudinal and transverse reinforcement ratios. Results for GFRP, steel and hybrid reinforcement configurations are provided. The results were collected from analysis output files of more than 60,000 columns, and tabulated in a form that is suitable for generating analytical strength curves. The provided data format allows the generation of strength curves for a wide range of slenderness ratios and the applied load eccentricities. Inspecting the analytical strength curves could provide insights on the slenderness limits for maintaining specific strength thresholds. Also, further investigations of data could provide a group of recommendations to avoid longitudinal and transverse reinforcement underutilization. Additional data processing could provide axial load-bending moment interaction diagrams for different columns` configurations taking into consideration the slenderness effects. The use of interaction diagrams in inspecting slender columns behavior is a ubiquitous subject that has been utilized in many recent research papers. Moreover, the results of a sensitivity analysis are provided within the article.

## Specifications Table

**Table:** 

Subject	Civil and Structural Engineering
Specific subject area	Circular columns analytical strength curves
Type of data	Table
How data were acquired	Collected from analysis results
Data format	Analyzed Filtered
Parameters for data collection	Longitudinal and transverse reinforcement ratios; slenderness ratios; reinforcement type and its strength
Description of data collection	Data were extracted from the analysis output files and were sorted and tabulated in .xlsx files with a formatting that allows the formulation of analytical strength curves. Moreover, sensitivity analysis results are provided within this paper.
Data source location	Institution: American University of Sharjah City/Town/Region: Sharjah Country: United Arab Emirates 25° 18′ 41.81 N″, 55° 29′ 33.53 E″
Data accessibility	Repository name: Zenodo Data identification number: 10.5281/zenodo.4568636 Direct URL to data: http://doi.org/10.5281/zenodo.4568636 Script used for data collection is provided with the article Other data is included in this article

## Value of the Data

• The sensitivity analysis data provided could be used for developing tornado charts, which are very efficient in evaluating the influence of different parameters on the column`s axial and bending load capacities. Sensitivity analysis can be described as the study that apportion different inputs according to their impact on a specific output [1]. Tornado charts are common in decision analysis [2], and are suitable for depicting sensitivity analysis results. Several research studies adopted using tornado charts in different domains, like retrofitting of concrete columns [3], earthquake engineering [4], and nonlinear finite element analysis of buckling restrained braces [5]. A typical tornado chart consists of horizontal bars called swings. Each swing represents the variation in model performance on using the upper and lower bounds of a specific parameter. The swings/parameters are sorted in a descending according to their length/impact, thus giving the diagram its tornado-like shape.
• Columns axial load capacity at different slenderness ratio values can provide an insight on the possibility of the columns` stability failure [6]. Moreover, insights on confined columns’ strength degradation due to slenderness could be derived to avoid confinement material underutilization. The given data could be used for assessing and proposing modifications to the design formulas of columns with GFRP or hybrid reinforcement. Similarly, recent research studies have utilized their available datasets to assess and propose new design guidelines [7], [8], [9], [10].
• The given data could be processed for developing axial load-bending moment interactions for circular slender columns.

## 1 Data Description

The data provided in the paper can be divided into two separate categories. The first one is a summary of a sensitivity analysis in which the impact of changing different input parameters was investigated. The investigated input parameters were the concrete compressive strength ($f_{cu}$), the longitudinal reinforcement ratio ($\rho_{l}$), and the transverse reinforcement ratio ($\rho_{t}$). While the reported output parameters were the confinement efficiency (CE), the axial load capacity (P), and the pending moment capacity (M). The sensitivity analysis data is provided for four groups of columns with the configurations given in Table 1. For each group, the upper and lower bound as well as the reference values of the input parameters were set as provided in Table 2. The sensitivity analysis outputs for the upper and lower bound cases are normalized and presented in Table 3. Given that each output value was normalized by relating to its reference-values counterpart.

**Table 1:** Properties of the sensitivity analysis groups.

Group	Longitudinal Reinforcement Material	Transverse Reinforcement Material
S1	GFRP	GFRP
S2	Steel	Steel
S3	Hybrid	GFRP
S4	Hybrid	Steel

**Table 2:** Assigned values to the sensitivity analysis parameters.

Parameter	Groups	Lower bound	Reference value	Upper bound
concrete compressive strength ($f_{cu}$) [MPa]	All	30	60	90
Longitudinal reinforcement ratio ($\rho_{l}$) [%]	All	1	4	7.7
Transverse reinforcement ratio ($\rho_{t}$) [%]	S1 and S3	2.7	5.9	9.4
	S2 and S4	5.6	7.8	9.4

**Table 3:** Sensitivity analysis outputs.

		$P/P_{reference}$ [%]		$M/M_{reference}$ [%]		$CE/CE_{reference}$ [%]	
Group	Parameter	Lower bound	Upper bound	Lower bound	Upper bound	Lower bound	Upper bound
S1	$f_{cu}$	94.40	106.13	97.55	102.42	161.62	79.46
	$\rho_{l}$	73.72	137.94	33.98	179.73	100.00	100.00
	$\rho_{t}$	68.26	121.00	99.75	101.36	73.61	118.32

S2	$f_{cu}$	84.30	128.87	86.80	110.88	159.85	80.05
	$\rho_{l}$	85.58	118.00	37.03	160.18	100.00	100.00
	$\rho_{t}$	95.31	106.73	100.00	100.00	88.18	107.07

S3	$f_{cu}$	92.02	112.68	96.92	103.60	161.62	79.46
	$\rho_{l}$	81.40	127.99	39.37	177.61	100.00	100.00
	$\rho_{t}$	81.35	122.03	100.19	102.97	73.61	118.32

S4	$f_{cu}$	92.24	117.34	95.74	103.20	159.85	80.05
	$\rho_{l}$	82.27	125.29	39.38	175.64	100.00	100.00
	$\rho_{t}$	86.53	107.95	97.45	100.71	88.18	107.07

**Fig. 1:**
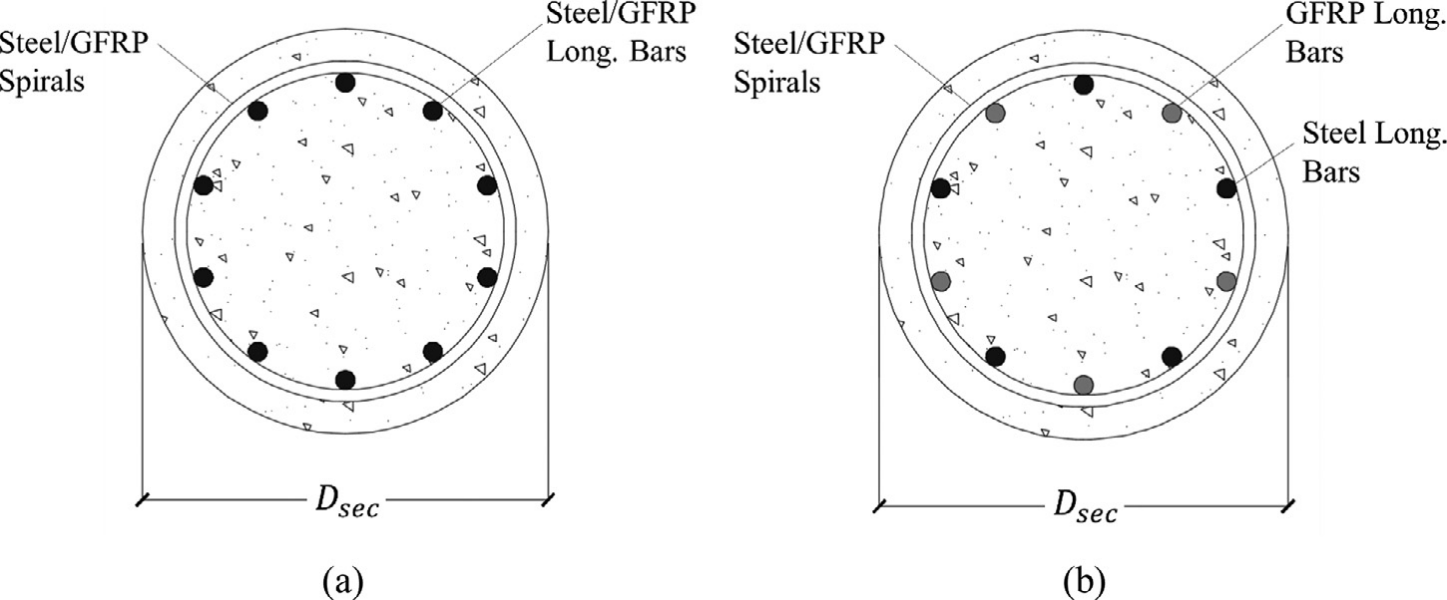
Different longitudinal reinforcement configurations (a) Steel or GFRP bars (b) Hybrid configuration.

**Fig. 2:**
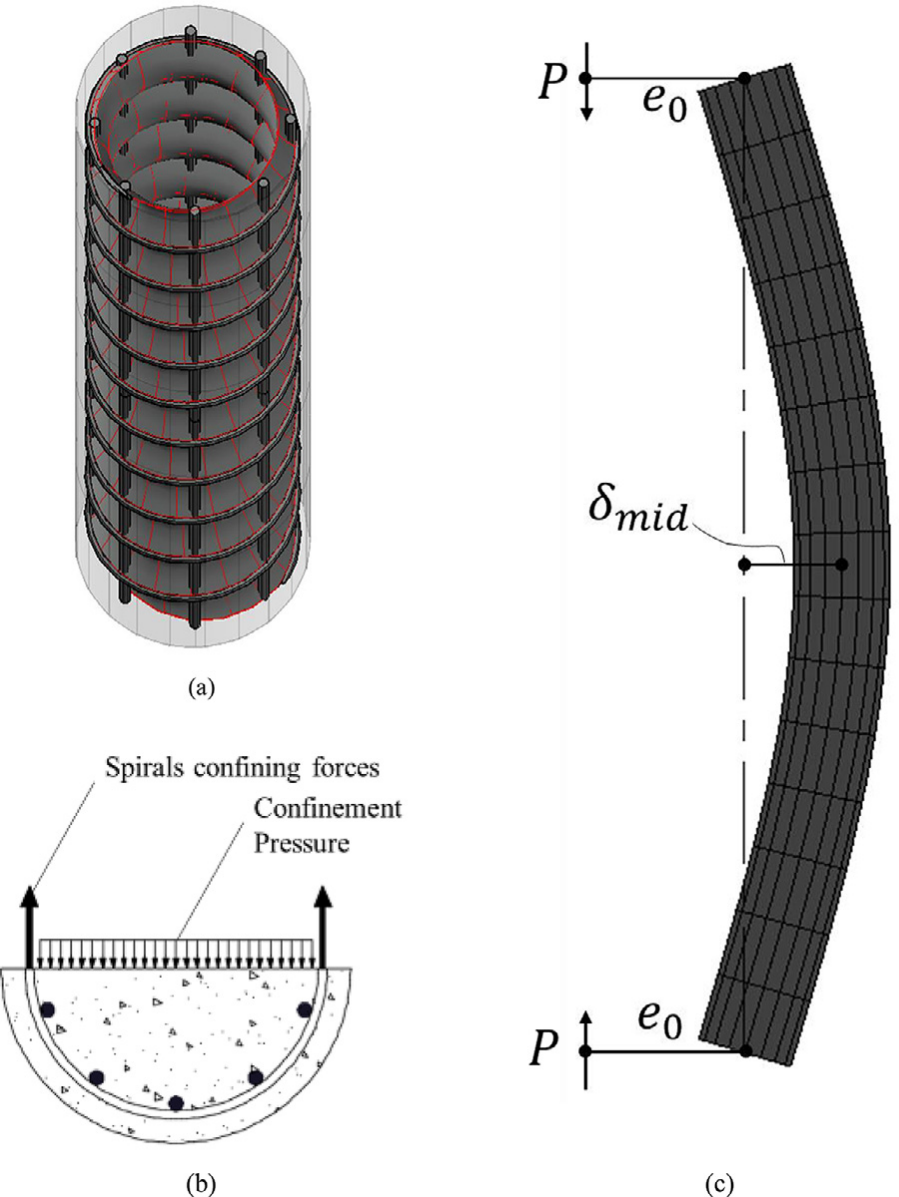
Different modelling considerations (a) The effectively confined core due to transverse reinforcement (b) Confinement pressure on the concrete core (c) Slenderness effects.

The data values presented in the second category (at the URL given in the “Data accessibility” section) are the columns axial load capacity including the slenderness effects as an independent parameter. Plotting the column's axial strength on the vertical axis versus the slenderness ratio on the horizontal axis yields the strength curve of that column for a specific loading configuration. Analytical strength curves were developed by integrating the analysis results from numerous output files. The analyzed columns were reinforced with a single layer of reinforcement. GFRP and steel rebars were used for transverse and longitudinal reinforcement. In some columns a hybrid longitudinal reinforcement configuration was investigated in which both GFRP and steel rebars were used, each representing half of the longitudinal reinforcement ratio, see Fig. 1. Lateral confinement was provided by either steel or GFRP spirals with different configurations yielding different transverse reinforcement ratios. Fig. 2 shows different modelling considerations represented implicitly within the results. The analysis results were collected and sorted into 30 groups with the properties given in Table 4.

**Table 4:** Group properties of the analytical results

				*GFRP*		*Steel*		

Group No.	Transverse Reinforcement Material	Transverse Reinforcement Ratio $\rho_{t}\;\%$	Longitudinal Reinforcement Material	$f_{fu}$ [MPa]	$E_{f}$ [GPa]	$f_{y}$ [MPa]	$E_{s}$ [GPa]	Data File
1	GFRP	3.0	GFRP	900	40	N/A	N/A	G01.xlsx
2	GFRP	6.0	GFRP	900	40	N/A	N/A	G02.xlsx
3	GFRP	9.0	GFRP	900	40	N/A	N/A	G03.xlsx
4	GFRP	3.0	GFRP	1000	50	N/A	N/A	G04.xlsx
5	GFRP	6.0	GFRP	1000	50	N/A	N/A	G05.xlsx
6	GFRP	9.0	GFRP	1000	50	N/A	N/A	G06.xlsx
7	GFRP	3.0	GFRP	1100	60	N/A	N/A	G07.xlsx
8	GFRP	6.0	GFRP	1100	60	N/A	N/A	G08.xlsx
9	GFRP	9.0	GFRP	1100	60	N/A	N/A	G09.xlsx
10	Steel	6.0	Steel	N/A	N/A	420	200	G10.xlsx
11	Steel	7.5	Steel	N/A	N/A	420	200	G11.xlsx
12	Steel	9.0	Steel	N/A	N/A	420	200	G12.xlsx
13	GFRP	3.0	Hybrid	900	40	420	200	G13.xlsx
14	GFRP	6.0	Hybrid	900	40	420	200	G14.xlsx
15	GFRP	9.0	Hybrid	900	40	420	200	G15.xlsx
16	GFRP	3.0	Hybrid	1000	50	420	200	G16.xlsx
17	GFRP	6.0	Hybrid	1000	50	420	200	G17.xlsx
18	GFRP	9.0	Hybrid	1000	50	420	200	G18.xlsx
19	GFRP	3.0	Hybrid	1100	60	420	200	G19.xlsx
20	GFRP	6.0	Hybrid	1100	60	420	200	G20.xlsx
21	GFRP	9.0	Hybrid	1100	60	420	200	G21.xlsx
22	Steel	6.0	Hybrid	900	40	420	200	G22.xlsx
23	Steel	7.5	Hybrid	900	40	420	200	G23.xlsx
24	Steel	9.0	Hybrid	900	40	420	200	G24.xlsx
25	Steel	6.0	Hybrid	1000	50	420	200	G25.xlsx
26	Steel	7.5	Hybrid	1000	50	420	200	G26.xlsx
27	Steel	9.0	Hybrid	1000	50	420	200	G27.xlsx
28	Steel	6.0	Hybrid	1100	60	420	200	G28.xlsx
29	Steel	7.5	Hybrid	1100	60	420	200	G29.xlsx
30	Steel	9.0	Hybrid	1100	60	420	200	G30.xlsx

The data collected for each group provide the strength-slenderness variation for given $\rho_{l}$ and $e_{o}/D_{sec}$ values. Where $e_{o}/D_{sec}$ is the ratio of the applied load eccentricity at the column`s edge to the diameter of the column's cross-section. The collected data of each group is given in a specific file as provided in Table 4. Within each file, the data values are tabulated in columns. Given that the first one lists the value of the slenderness ratio (KL/r) for each data row, while the other columns provide the strength values. Each data column represents a strength curve, and its header provides the values of $\rho_{l}$ and $e_{o}/D_{sec}$ for that curve. For example, a curve with header “Rho4_e/D0.05” means that the value of $\rho_{l}=4\;\%$ and $e_{o}/D_{sec}=0.05$.

**Fig. 3:**
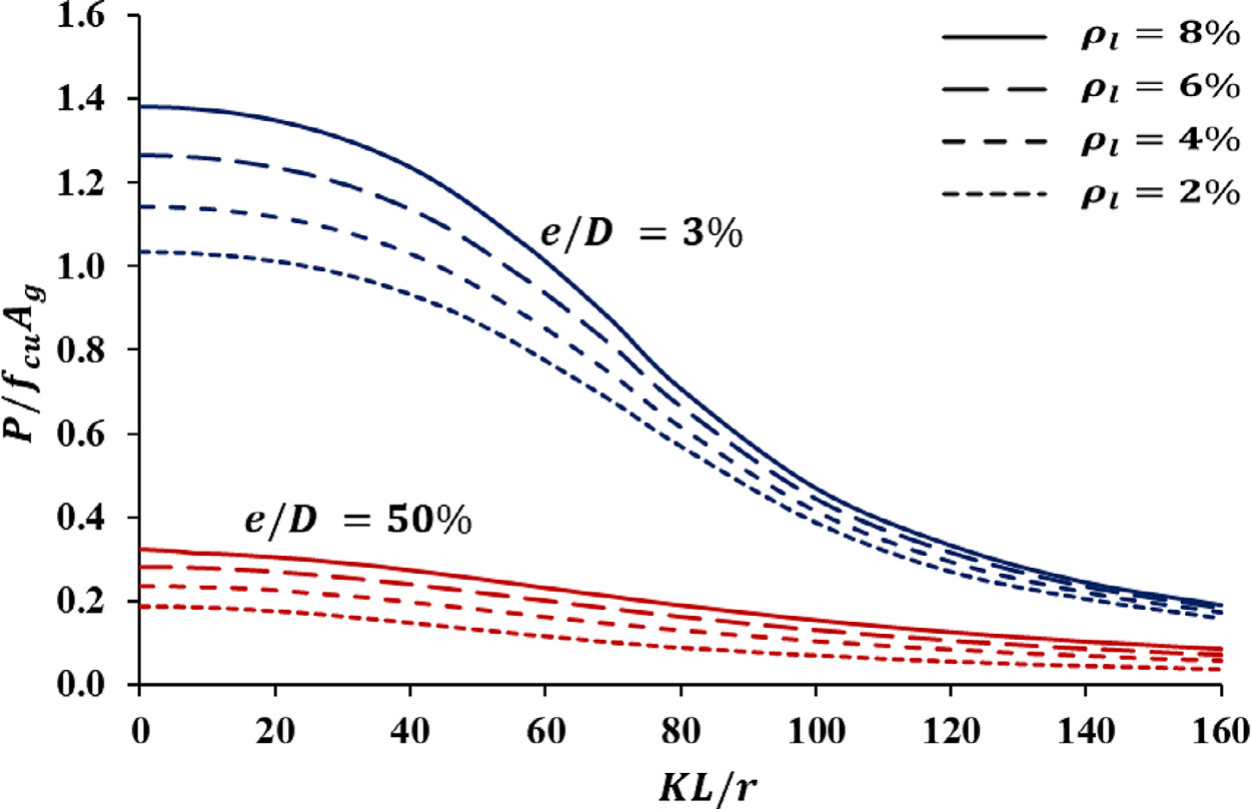
A typical analytical strength curve.

At low values of slenderness ratio, the columns are referred to as “short columns” in which the column behavior is generally governed by the concrete and the longitudinal reinforcement strength. Increasing the amount of the transverse reinforcement enhances the confinement effects, thus introducing more axial load capacity to the column`s cross-section. As the value of the column`s slenderness increases, the axial load capacity decreases due to the increase of the buckling-induced additional moment. After exceeding a specific slenderness limit, the columns start undergoing an elastic buckling which is generally governed by the elastic modulus and moment of inertia of the columns` cross-section [11]. Given the provided data, strength curves of different reinforcement configurations and load eccentricities could be plotted against each other for comparisons and data interpretation. Fig. 3 provides a sample of a strength curves chart that could be generated for two eccentricity values from one group of the provided ones.

Finally, it should be noted that the strength values were presented in a normalized form $P/f_{cu}A_{g}$, where $A_{g}$ is the gross area of the column`s cross-section. Moreover, the provided data could be used for generating axial load-bending moment interaction diagrams for short and slender columns. The interaction diagrams are, typically, plots of the axial load capacity on the vertical axis versus the bending moment capacity on the horizontal axis. The normalized bending moment capacities for the provided column`s data could be derived by multiplying each normalized axial load capacity $P/f_{co}A_{g}$ by its corresponding eccentricity ratio $e_{o}/D_{sec}$.

## 2 Experimental Design, Materials and Methods

GFRP bars have a long elastic range with high ultimate strength which could enhance columns’ axial-load and bending-moment capacities. While steel bars provide larger stiffness to the columns through early loading stages due to its relatively higher elastic modulus. Integrating both types of reinforcement could alter the behavior of columns through different loading stages. Hybrid reinforcement configurations have been studied in different research studies [12], [13], [14], [15], [16], [17], and in different cases the hybrid reinforcement configurations outperformed those of steel or GFRP only**.** Thus, within this paper, different hybrid/non-hybrid reinforcement configurations were introduced to cover a wide range of reinforcement possibilities.

The use of concrete and reinforcement bars with high strength values, and introducing new confinement techniques encourage reducing the columns' diameter. Which in return yields more slender columns and makes them more susceptible to capacity loss due to buckling effects. Several researches have adopted the use of strength curves for determining adequate design recommendations [10,[18], [19], [20]. Several analytical modeling approaches were proposed to consider the slenderness effects using second order analysis [6,^9^,^12^,[20], [21], [22], [23], [24], [25]. Using these approaches, an integrated model was developed to perform the required analytical simulations.

**Fig. 4:**
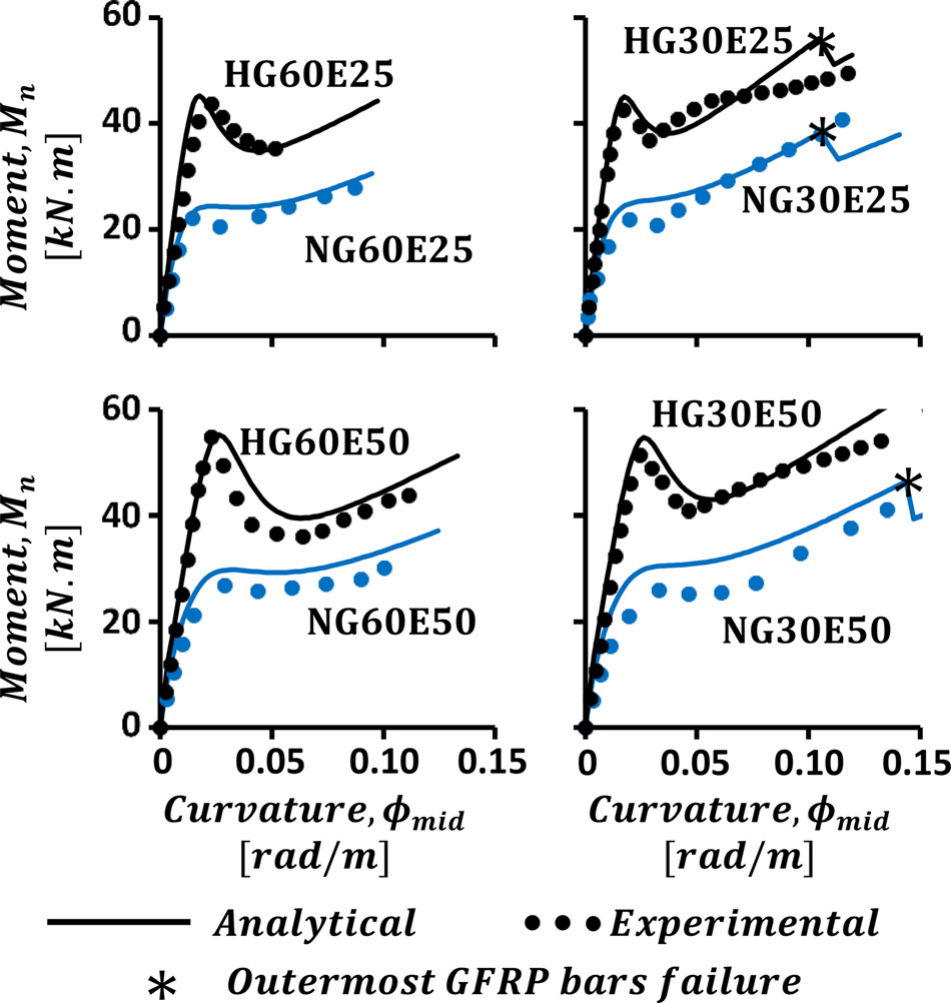
Verification against experimental results of Hasan et al. [21].

The model was verified against an experimental dataset provided by Hasan et al. [21], which summarizes the results of experimental investigations conducted on normal and high strength columns by Hadi et al. [26,27], respectively. The dataset included the results for 8 columns reinforced with GFRP longitudinal bars and transverse helices. A concrete mix with a compressive strength of 37 MPa was used for the normal strength columns, while that used for high strength columns was attributed with an 85 MPa compressive strength. Each tested specimen was assigned a descriptive name composed of 3 sections. For example, “NG60E25” denotes a specimen with normal strength concrete (“NG”) with helix pitch of 60 mm and an applied axial load eccentricity of 25 mm. The names of specimens with high strength concrete started with the section “HG”. All the tested specimens had a 210 mm diameter and 800 mm length. A longitudinal reinforcement of 6 No. 4 GFRP bars was adopted for all the columns, while the transverse reinforcement consisted of a No. 3 GFRP helix with a pitch that varies from one specimen to the other according to the adopted naming convention. Fig. 4 shows a comparison between the experimental moment-curvature curves and the analytical ones computed using the developed analytical model. Using the stress-strain relations proposed by Hasan et al. [21], the analytical results have shown an acceptable correlation with the experimental ones.

The developed model was used to analyze more than 60,000 columns under different loading conditions and an object-oriented based Python script was written for collecting data from the analysis output files. Each output files included the axial load capacities for one column configuration, including KL/r, with different $e_{o}/D_{sec}$ values. Two classes were created to represent data holders throughout the code execution. The first class “DataFile” was developed to open, read, and store the data found in an output folder. The “DataFile” object’ constructor uses the file path and the required $e_{o}/D_{sec}$ as input parameters. Then, it searches for the axial load capacity for the given $e_{o}/D_{sec}$ values within the file. After finding the required values, they are stored as object attributes for further use. The second class “KLrCurve” was used a data holder for one strength curve. Within each curve, the axial strength values for a specific column configuration with one loading eccentricity are stored as a function of KL/r.

The script starts by collecting data for a specific group of output files and storing their results in different “DataFile” instances. Then, for each $e_{o}/D_{sec}$ value, the script generates different “KLrCurve” instances using the data stored in the “DataFile” ones. Given the required parameters per each data group, the script collects the appropriate “KLrCurve” instances and prints their results in .xlsx files. The data values are printed in an explicit form that allows, directly, creating charts of strength curves. Finally, the collected data files were inspected to filter out any odd values.

## Ethics Statement

This data article is in full compliance with the ethical requirements for publication in *Data in Brief.*

## CRediT Author Statement

**Mohammad AlHamaydeh:** Conceptualization, Methodology, Investigation, Writing – review & editing, Supervision, Project administration; **Fouad Mostafa Amin:** Methodology, Investigation, Data curation, Writing – original draft, Writing – review & editing, Visualization.

## Declaration of Competing Interest

The authors declare that they have no known competing financial interests or personal relationships which have or could be perceived to have influenced the work reported in this article.

## Appendix Supplementary materials

Image, application 1
